# The complete plastid genome sequence of *Chloranthus fortunei* (A. Gray) Solms-Laub. in Chloranthaceae

**DOI:** 10.1080/23802359.2022.2132840

**Published:** 2022-10-25

**Authors:** Jong-Soo Kang, Bo-Yun Kim, Ki-Oug Yoo

**Affiliations:** a Department of Biological Sciences, Kangwon National University, Chuncheon, South Korea; b Plant Resources Division, National Institute of Biological Resources, Incheon, South Korea

**Keywords:** *Chloranthus fortunei*, *Chloranthus*, Chloranthaceae, plastid genome

## Abstract

*Chloranthus fortunei* (A. Gray) Solms-Laub. is a perennial herb in a basal angiosperm family Chloranthaceae. Here, we reported the complete plastid genome of *C. fortunei* using Illumina short-read data. The total genome size was 157,063 bp in length, containing 79 protein-coding genes, 30 tRNA genes, and four rRNA genes. The gene content and order were consistent with previously reported *Chloranthus* plastid genomes. The overall GC content of the *C. fortunei* plastid genome was 39.0%. In the phylogenetic result, genus *Chloranthus* was monophyletic and divided into two subclades: *C. japonicus*+*C. angustifolius*+*C. fortunei*, and *C. henryi*+*C. spicatus*+*C. erectus*. Our phylogenetic result was consistent with previous phylogenetic studies, and was supported by a previously proposed infrageneric classification of the genus *Chloranthus*.

*Chloranthus* Swartz (Chloranthaceae) consists of two subgenera, subgenus *Tricercandra* and subg. *Chloranthus*, based on androecium morphology, such as the extent of splitting in the tripartite lobes (Kong 2000a; Kong and Chen 2000; Kong et al. 2002). *Chloranthus fortunei* (A. Gray) Solms-Laub. (1869) belongs to subg. *Tricercandra*, and is distributed in southern parts of China, Korea, and Japan (Kim 2007; Xia and Jérémie 2007). This species has been cultivated as an ornamental herb, and also used for the Chinese folk medicine as a treatment of bone fractures (Ben Cao 1999). Morphologically, *C. fortunei* is very similar to *C. japonicus* Siebold which is widely distributed in East Asia (Kim 2007; Xia and Jérémie 2007); however, *C. fortunei* can be distinguished from the former by the anther position of the androecium, ploidy level, and tripartite androecium with long longitudinal connections (Kong 2000b; Kim 2007; Xia and Jérémie 2007; Figure 1). Whole plastid genomes have been widely used for molecular phylogenetics, species identifications, and conservation genetics (Burke et al. 2012; Huang et al. 2014; Walker et al. 2014). Here, we report the plastid genome of *C. fortunei*, which will be useful for the conservation genetic studies of this species as well as phylogenetic reconstructions of *Chloranthus* and other basal angiosperms.

**Figure 1.**
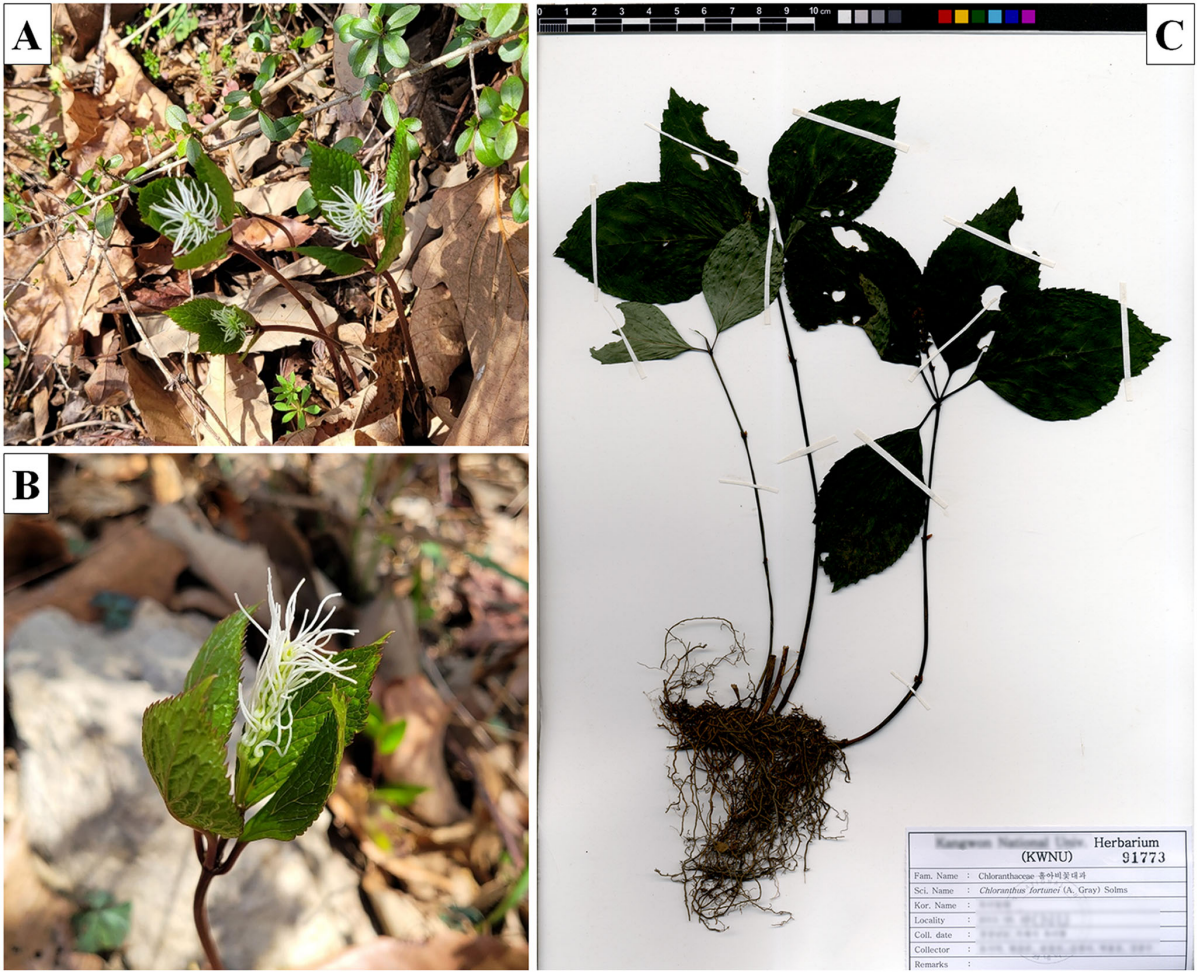
*Chloranthus fortunei*. This species has a tripartite androecium with long longitudinal connections. (A) Habitat, (B) flower, and (C) the specimen deposited in Kangwon National University Herbarium (KWNU) under the voucher no. KWNU91773. The photos of *C. fortunei* in field (A, B) and the voucher specimen (C) were taken and provided by Jong-Soo Kang and Ki-Oug Yoo, respectively.

Leaf material of *C. fortunei* was collected from Ongnyeobong, Geoje-si, Gyeongsangnam-do province of South Korea (latitude 34.8455, longitude 128.6954). The voucher specimen (KWNU91773) has been deposited in the Kangwon National University Herbarium (KWNU; https://biology.kangwon.ac.kr/, Ki-Oug Yoo, yooko@kangwon.ac.kr). Total genomic DNA was extracted from silica gel dried leaves using the Exgene Plant SV Midi Kit (Geneall Biotechnology, Seoul, South Korea). Paired-end reads of 2 × 150 bp were generated using an Illumina HiSeq Xten (Theragen Bio Co. Ltd., Suwon, South Korea). A total of 2.26 GB raw reads of 150 bp were generated, of which 146,514 paired-end reads were extracted as plastid genome sequences using a reference genome sequence of the *C. japonica* plastid genome (KP256024). Using 146,514 reads, the *de novo* assembly was performed using GetOrganelle pipeline (Jin et al. 2020) with *C. japonica* plastid genome as a reference, and the assembled contig was manually confirmed using Geneious 7.1 (Biomatters Ltd, Auckland, New Zealand). The initial annotation of the *C. fortunei* plastid genome was performed using GeSeq (Tillich et al. 2017). After the initial annotation, putative starts, stops, and intron positions were determined by comparison with homologous genes in previously reported *Chloranthus* plastid genomes. The tRNA genes were annotated using GeSeq and tRNAscan-SE (Schattner et al. 2005). The annotated sequence was deposited in the NCBI GenBank under accession number ON023121, and the circular map of the *C. fortunei* plastid genome was drawn using the CPGview (http://www.1kmpg.cn/cpgview/).

The genome size of the *C. fortunei* plastid genome was 157,063 bp, including a pair of inverted repeat (IR) regions of 26,102 bp separated by the small single-copy (SSC) region of 18,484 bp, and the large single-copy (LSC) region of 86,375 bp (Figure 2). The *C. fortunei* plastid genome contained 113 genes, 18 of which were duplicated in the IR region, giving a total of 131 genes. The plastid genome of *C. fortunei* contained 30 distinct tRNAs, seven of which were duplicated in the IR region. Ten protein-coding genes (*atpF*, *ndhA*, *ndhB*, *petB*, *petD*, *rps12*, *rps16*, *rpl2*, *rpl16*, and *rpoC1*) and six tRNA genes (*trnA*-*UGC*, *trnG*-*GCC*, *trnI*-*GAU*, *trnK-UUU*, *trnL-UAA*, and *trnV-UAC*) contained one intron, while two genes (*clpP*, *ycf3*) contained two introns. A trans-spliced *rps12* gene was divided into two independent transcription units (exon 1, and exons 2–3) as described in previous studies (Hildebrand et al. 1988; Schmitz-Linneweber et al. 2006). The overall GC content was 39.0% in the entire genome, 37.4% in the LSC, 43.2% in the IR, and 34.1% in the SSC regions.

**Figure 2.**
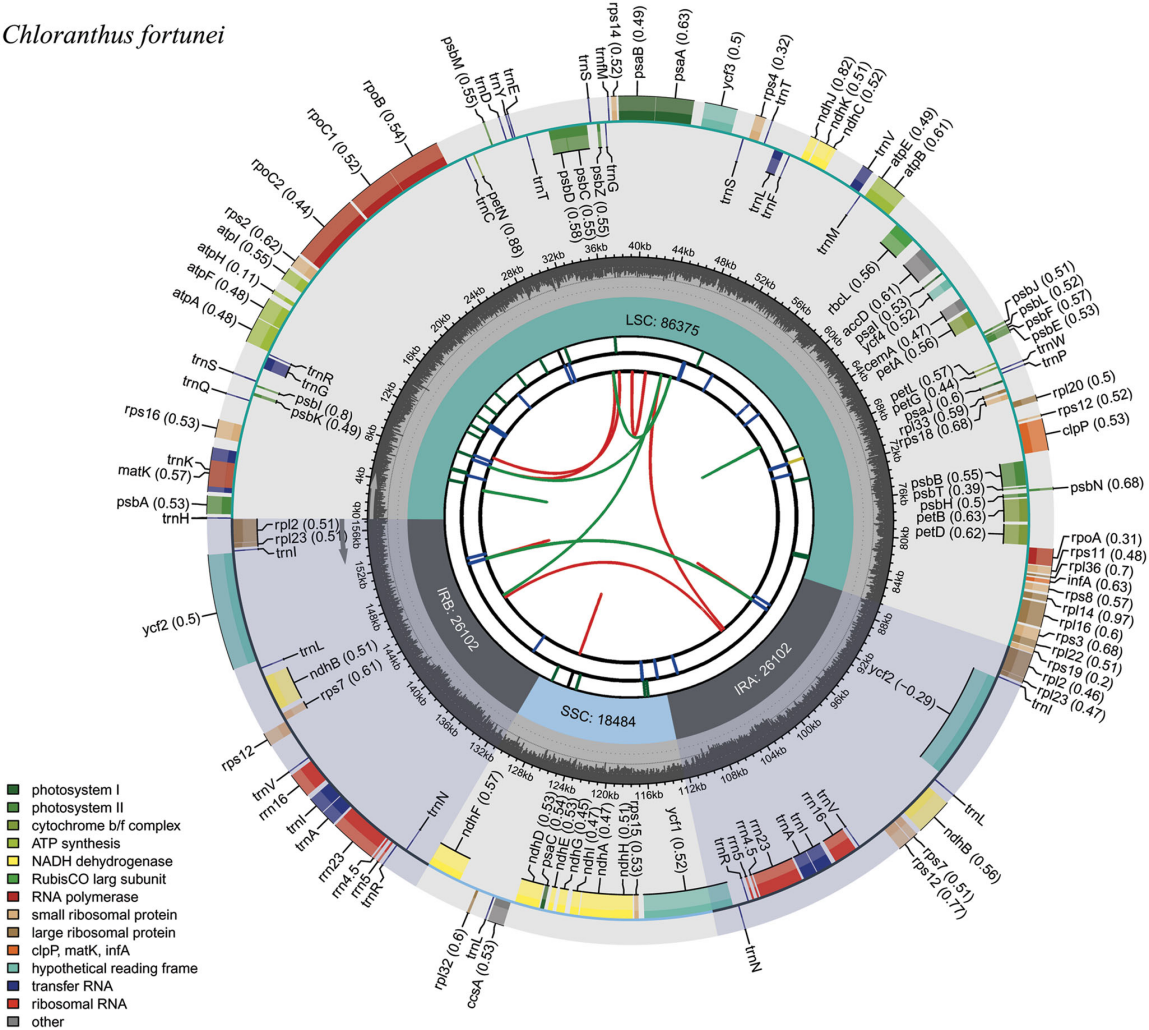
The map of the *Chloranthus fortunei* plastid genome. The circular map of the *C. fortunei* plastome was drawn using the CPGview program. The map consists of six circles and information about each circle is as follows: (from the center) the first circle indicates repeat distribution. The second circle indicates the tandem repeats with short bars. The third circle indicates the microsatellite sequences with short bars. The fourth circle indicates the size of LSC, SSC, and IR regions. The fifth circle indicates the GC content. The sixth circle indicates the genes having different colors based on their functions.

Phylogenetic analysis based on 78 protein-coding genes was performed using representative species from Amborellales in basal angiosperms to Magnoliales in magnoliids, and *Amborella trichopoda* was selected as the outgroup (Figure 3). A total of 69,404 bp was aligned using MAFFT (Katoh and Standley 2013). Maximum-likelihood (ML) analysis was performed using RAxML v. 7.4.2 with 1000 bootstrap replicates and the GTR + I+G model (Stamatakis 2006; Darriba et al. 2012). Our phylogenetic result was consistent with topologies from previous studies in which all families and orders were monophyletic (Angiosperm Phylogeny Group 2016) (Figure 1). Within Chloranthaceae, *Sarcandra glabra* was sister to the clade of *Chloranthus* with 100% bootstrap supporting values, and the genus *Chloranthus* was monophyletic as shown in previous studies (Kong et al. 2002; Zhang et al. 2011). The three species, *C. fortunei*, *C. angustifolius*, and *C. japonicus* of subg. *Tricercandra* formed a subclade, and the subclade was sister to the other clade of subg. *Chloranthus* including *C. henryi*, *C. spicatus*, and *C. erectus* with 100% bootstrap supporting values (Figure 3). The pairwise identity of concatenated 78 protein-coding gene sequences within the genus *Chloranthus* was 99.2%, and those within both two subgenera was 99.5%, respectively.

**Figure 3.**
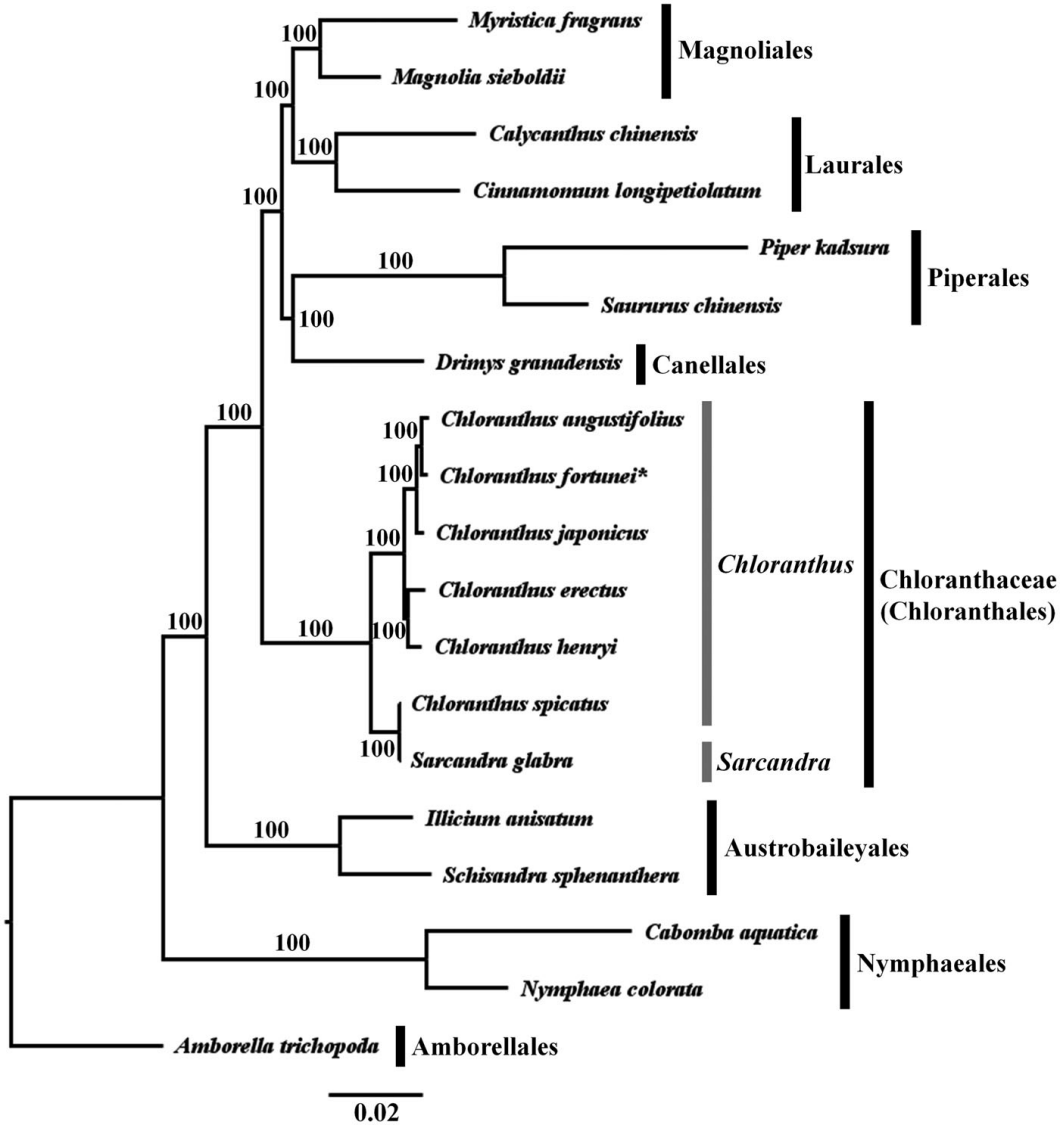
Phylogenetic tree based on 78 protein-coding genes using the ML method. Asterisk indicates newly reported plastid genome in this study. Bootstrap values are shown near the nodes. The following sequences were used: *Myristica fragrans* MN495963 (Cai et al. 2021), *Magnolia sieboldii* MN990583 (Wang et al. 2020), *Cinnamomum longipetiolatum* MN698965 (Zheng et al. 2019), *Calycanthus chinensis* MG561304 (Chen et al. 2019), *Piper kadsura* KT223569 (Lee et al. 2016), *Saururus chinensis* MN263891 (Jin et al. 2019), *Drimys granadensis* DQ887676 (Cai et al. 2006), *Chloranthus fortunei* ON023121 (this study), *Chloranthus angustifolius* MW581013 (Zhang unpublished), *Chloranthus japonicus* KP256024 (Sun et al. 2016), *Chloranthus erectus* MH394412 (Zeng et al. 2018), *Chloranthus spicatus* EF380352 (Hansen et al. 2007), *Chloranthus henryi* MK922064 (Liu et al. 2019), *Sarcandra glabra* MH939147 (Wang et al. 2020), *Schisandra sphenanthera* MK193856 (Wei et al. 2020), *Illicium anisatum* KY085919 (Zhang and Handy unpublished), *Cabomba aquatica* MG720559 (Gruenstaeudl et al. 2018), *Nymphaea colorata* MT107631 (Sun et al. 2021), and *Amborella trichopoda* AJ506156 (Goremykin et al. 2003).

## Data Availability

The genome sequence data that support the findings of this study are openly available in GenBank of NCBI (https://www.ncbi.nlm.nih.gov/) under the accession no. ON023121. The associated BioProject, SRA, and Bio-Sample numbers are PRJNA816642, SRR18360190, and SAMN26686909, respectively.
